# Effects of the mTOR inhibitor Rapamycin on Monocyte-Secreted Chemokines

**DOI:** 10.1186/s12865-014-0037-0

**Published:** 2014-09-26

**Authors:** Hugo You-Hsien Lin, Kai-Ting Chang, Chi-Chih Hung, Chang-Hung Kuo, Shang-Jyh Hwang, Hung-Chun Chen, Chih-Hsing Hung, Sheng-Fung Lin

**Affiliations:** Division of Nephrology, Kaohsiung Medical University Hospital, Kaohsiung Medical University, Kaohsiung, Taiwan; Division of Hematology and Oncology, Department of Internal Medicine, Kaohsiung Medical University Hospital, Kaohsiung Medical University, 100 Tzyou First Road, Kaohsiung, 807 Taiwan; Department of Pediatrics, Kaohsiung Medical University Hospital, Kaohsiung Medical University, 100 Tzyou First Road, Kaohsiung, 807 Taiwan; Department of Internal Medicine, Kaohsiung Municipal Ta-Tung Hospital, Kaohsiung Medical University, Kaohsiung, Taiwan; Department of Pediatrics, Kaohsiung Municipal Ta-Tung Hospital, Kaohsiung Medical University, Kaohsiung, Taiwan; Department of Pediatrics, Kaohsiung Municipal Hsiao-Kang Hospital, Kaohsiung Medical University, Kaohsiung, Taiwan; Graduate Institute of Medicine, College of Medicine, Kaohsiung Medical University, Kaohsiung, Taiwan; Faculty of Renal Care, Kaohsiung Medical University, Kaohsiung, Taiwan; Faculty of Medicine, College of Medicine, Kaohsiung Medical University, Kaohsiung, Taiwan

**Keywords:** mTOR, Chemokine, Glomerulonephritis

## Abstract

**Background:**

Mammalian target of rapamycin (mTOR) inhibitors, such as sirolimus and its derivative, everolimus, are potent immunosuppressive and antiproliferative drugs. Inflammatory diseases are characterized by immunological dysfunction, and monocyte recruitment underlies the mechanism of cell damage. Chemokines attract inflammatory cells to sites of inflammation. Interleukin-8 (IL-8/CXCL8); the monocyte chemoattractant protein-1 (MCP-1/CCL2); the regulated on activation, normal T cell expressed, presumably secreted protein (RANTES/CCL5); the macrophage inflammatory protein (MIP)-1α (CCL3); and MIP-1β (CCL4) are involved in the pathogenesis of inflammation. However, whether mTOR inhibitors moderate the production of chemokines in monocytes remains unclear.

**Methods:**

A human monocyte cell line, THP-1, and primary monocytes obtained from human volunteers, were stimulated using lipopolysaccharide (LPS), and then treated with sirolimus. The expression of the MCP-1, RANTES, IL-8, MIP-1α, MIP-1β, and TNF-α proteins was measured using enzyme-linked immunosorbent assays, and intracellular signalling was examined using western blotting.

**Results:**

Sirolimus significantly suppressed the LPS-induced expression of MCP-1, IL-8, RANTES, MIP-1α, and MIP-1β in the THP-1 cells and human primary monocytes. The mitogen-activated protein kinase (MAPK) inhibitors that were examined suppressed the LPS-induced expression of MCP-1, IL-8, RANTES, MIP-1α, and MIP-1β. In addition, sirolimus suppressed the LPS-induced phosphorylation of p38 and p65 in the THP-1 and human primary monocytes.

**Conclusion:**

Sirolimus downregulates the expression of chemokines in monocytes, including MCP-1, RANTES, IL-8, MIP-1α, and MIP-1β, by inhibiting the NF-κB-p65 and MAPK-p38 signalling pathways.

## Background

Mammalian target of rapamycin (mTOR) is critical to cell differentiation, migration, and survival [1]. Inhibitors of mTOR, such as sirolimus or everolimus, have exhibited antiinflammatory, antifibrotic, antitumor, and antifungal properties, suggesting that mTOR signalling is involved in various cellular functions [2]. Activation of mTOR phosphorylated p70 ribosomal S6kinase and eukaryotic initiation factor-4E leads to cell hypertrophy, macrophage, T cell proliferation, and infiltration [1]. Recently, mTOR inhibitors have been applied to anticancer therapy [3] to prevent restenosis of the coronary arteries after angioplasty [4], and used in clinical trials and research pertaining to the tuberous sclerosis complex [5] and Alzheimer’s disease [6]. In kidney disease, although mTOR inhibitors are limited by the risk of exacerbating preexisting proteinuria [7], possibly attributable to inhibiting the vascular endothelial growth factor [8], mTOR has ameliorated the tubulointerstitial disease associated with chronic proteinuria in experimental animal models and decreased proteinuria values in patients with steroid-resistant nephrotic syndrome [9,10].

Monocytes, which can differentiate into macrophages and dendritic cells, contribute to the pathogenesis of inflammation, an vital defence mechanism used by diseases, by secreting cytokines and chemokines, recruiting and activating leukocyte subsets that play various roles in inflammation by interacting with chemokine receptors [11]. Monocyte chemoattractant protein-1(MCP-1)/CCL2; chemokine (C-X-C motif) ligand 3 (CXCL3); the regulated on activation, normal T cell expressed, and presumably secreted protein (RANTES)/CCL5; macrophage inflammatory protein (MIP-1α)/CCL3; MIP-1β/CCL4; interleukin-8 (IL-8)/CXCL8; TNF-α; and corresponding receptors are involved in monocyte recruitment during inflammation [12]. In clinical applications, serum or urinary levels of these chemokines and expression in disease tissue could serve as biomarkers of disease diagnosis, prognosis, or treatment responses [13-16].

However, few studies have investigated the effect mTOR inhibitors exert on the expression of these chemokines. We hypothesized that mTOR inhibitors modulated these chemokines in monocytes, and clarified the detailed intracellular pathway mechanisms by which modulation occur, including mitogen-activated protein kinase (MAPK) and nuclear factor κB (NF-κB). We designed a series of experiments to test and verify our hypothesis.

## Methods

### Cell preparation

A human monocyte cell line, THP-1 (American Type Culture Collection, Rockville, MD, USA), was cultured in an RPMI 1640 medium (Sigma-Aldrich, St. Louis, MO, USA) supplemented with 10% foetal bovine serum, 100 U/mL of penicillin, and 100 μg/mL of streptomycin at 37°C in 5% CO_2_ in a humidified incubator. The THP-1 cells were collected by centrifugation, and resuspended in a fresh RPMI medium. Twenty-four well plates were seeded with 10^6^ cells/mL and incubated for 24 h. In preparation for the human primary monocyte experiments, peripheral blood samples were collected from 3 healthy volunteers after we obtained informed consent. The volunteers had no personal or family history of allergies. This study was approved by the Institutional Review Board of Kaohsiung Medical University Hospital (KMUH-IRB-20130333).

The blood samples were diluted with an equal volume of phosphate-buffered saline. Peripheral blood mononuclear cells (PBMCs) were isolated using density-gradient centrifugation (Lymphoprep, Oslo, Norway). Primary monocytes were isolated from the other PBMCs by using magnetically activated cell sorting involving an anti-CD 14 monoclonal antibody (Milteny Biotec, Bergisch Gladbach, Germany). The cells were stimulated using 0.2 μg/mL of lipopolysaccharide (LPS; Sigma-Aldrich) for 2 h before being treated using 0, 1, 5, or 10 ng/mL of sirolimus (Sigma-Aldrich). The cell supernatants were collected after 24 and 48 h.

### Cell viability assay

After LPS stimulation, the THP-1 cells were treated using 1, 5, or 10 ng/mL of sirolimus for 24 h, and cell viability was assessed using the WST-1 Cell Viability and Proliferation Assay (Roche Diagnostics, Indianapolis, IN, USA).

### Quantification of chemokine expression

The intracellular levels of MCP-1, IL-8, RANTES, MIP-1α, MIP-1β, and TNF-α proteins in the cell supernatants were determined using a commercially available enzyme-linked immunosorbent assay (ELISA) kit (R&D System, Minneapolis, MN, USA). The optical density of the ELISA samples was measured at 450 and 540 nm using a Dynatech MRX plate reader (Dynatech Laboratories, Chantilly, VA, USA), and the ELISA data were analysed using Revelation software (Westwood, NJ, USA).

### Mitogen-activated protein kinase and nuclear factor-kappa B assay

The THP-1 cells were treated for 1 h using 1 of 3 MAPK inhibitors: PD 98059, SB203580, or SP600125 (Sigma-Aldrich); the NF-κB inhibitor, BAY 11–7085; or the vehicle control. The cells were stimulated using 0.2 μg/mL of LPS for 48 h, and then the cell supernatants were collected for ELISA analysis.

### Western blot analysis

The THP-1 cells were stimulated using 0.2 μg/mL of LPS for 1 h and treated with 0, 5, or 10 ng/mL of sirolimus for 2 h. The cells were lysed using an equal volume of ice-cold lysis, and centrifuged at 13 000 × g for 15 min. The total amount of protein in the cell-lysate supernatants was determined using the BCA Protein Assay Reagent (Thermo-Fisher, Waltham, MA, USA). Cell-lysate samples were prepared using equivalent total protein concentrations, and analysed by employing western blotting. The blots were probed using primary antibodies (Santa Cruz Biotechnology, Dallas, TX, USA) generated against the following proteins: p38, signal regulated kinase (ERK), c-Jun N-terminal kinase (JNK), phosphorylated (phospho)-p38, phospho-ERK, phospho-JNK, NF-κB (p65), and phospho-p65. Primary antibody reactivity was visualised using a horseradish peroxidase-conjugated secondary antibody and an enhanced chemiluminescence system (GE Healthcare Life Sciences, Waukesha, WI, USA).

### Statistical analyses

Each experiment was replicated 6 times, and the data were presented as the mean ± standard deviation. Differences between the experimental and control groups were analysed using the Mann–Whitney *U*-test, and *P* < .05 was considered to indicate a statistically significant intergroup difference.

## Results

### Sirolimus did not reduce the viability of the THP-1 cells

**Figure 1:**
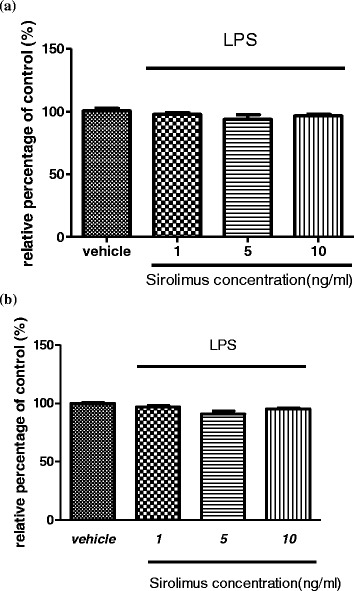
**In cell viability tests, treatment with sirolimus in concentrations of 1, 5, and 10 ng/mL did not affect cell viability in the THP-1 cells (a) and human monocytes (b).**

The 24-h sirolimus treatment did not significantly change the viability of the THP-1 cells and primary monocytes, compared with the control group (Figure 1 and b).

### Sirolimus suppressed lipopolysaccharide-induced chemokine expression in THP-1 cells and human primary monocytes

**Figure 2:**
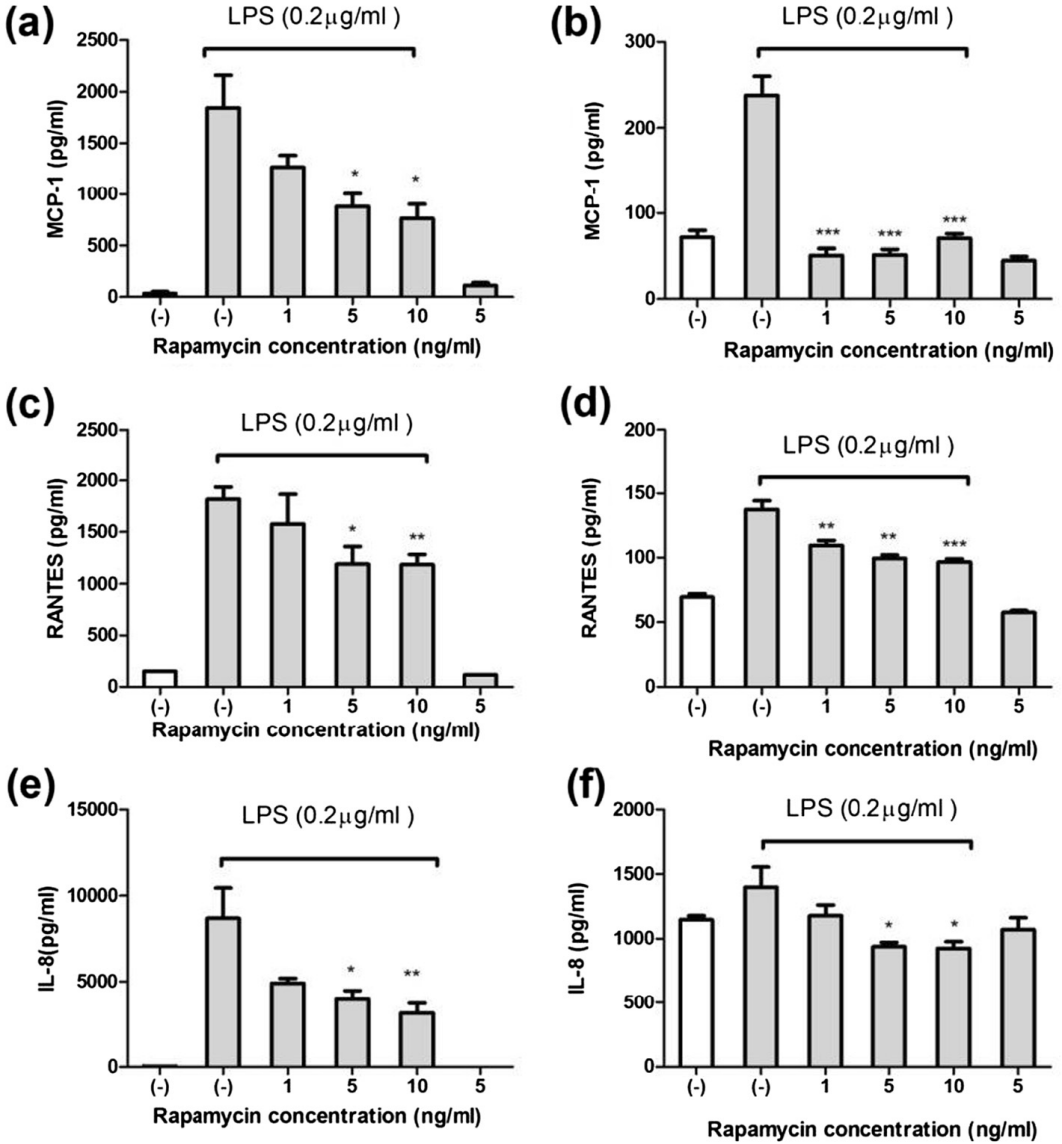
Sirolimus suppressed LPS-induced MCP-1, RANTES, and IL-8 production in THP-1 cells (a), (c), and (e), and primary human monocytes (b), (d), and (f) after 24 h of LPS stimulation (** ***P*** < .01 and *** ***P*** **< .005 between groups of LPS only and LPS plus sirolimus treatment).**

**Figure 3:**
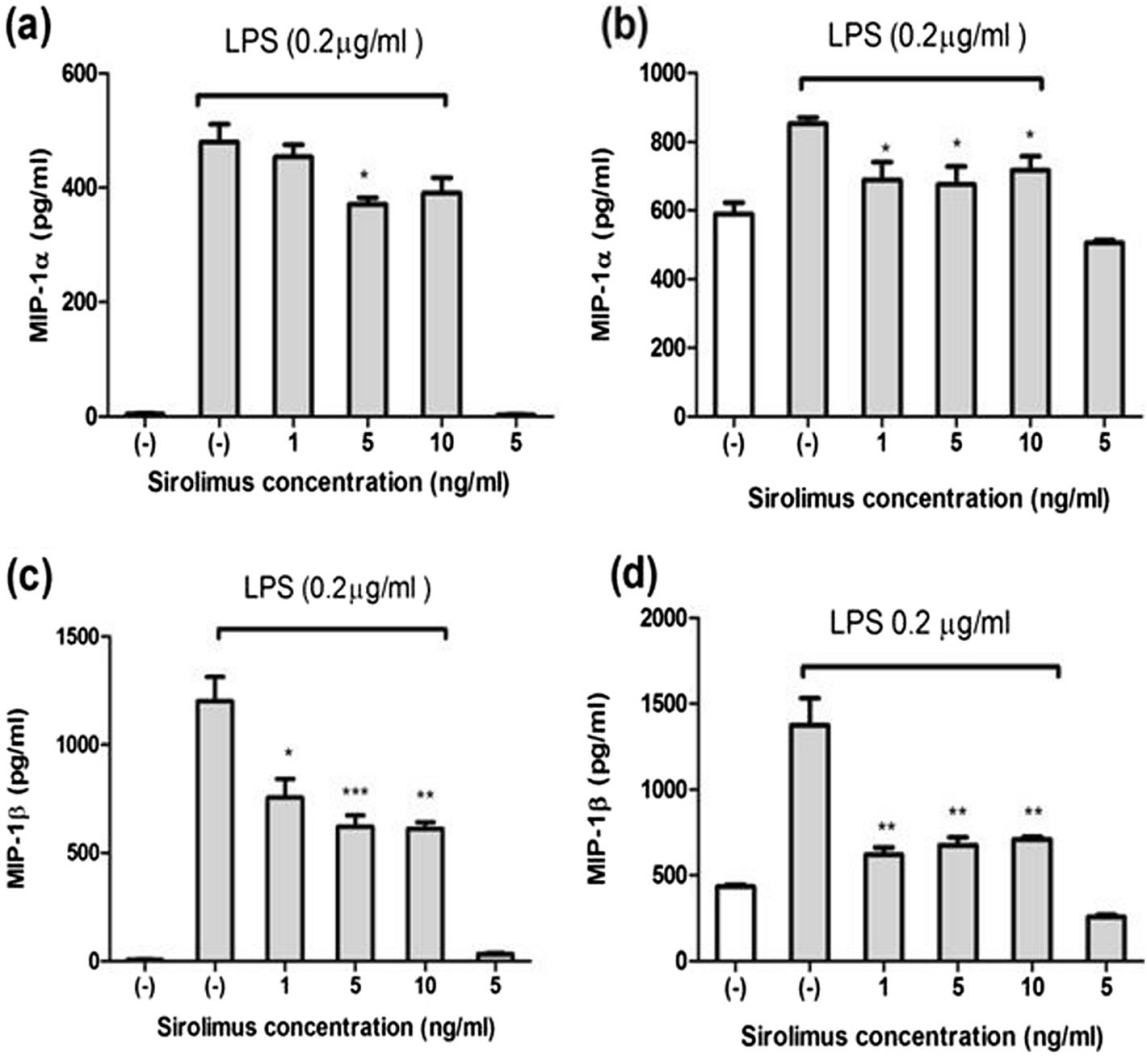
Sirolimus suppressed LPS-induced MIP-1α, and MIP-1β production in THP-1 cells (a) and (c), and primary human monocytes (b) and (d) after 24 h of LPS stimulation (** ***P*** < .01 and *** ***P*** **< .005 between groups of LPS only and LPS plus sirolimus treatment).**

**Figure 4:**
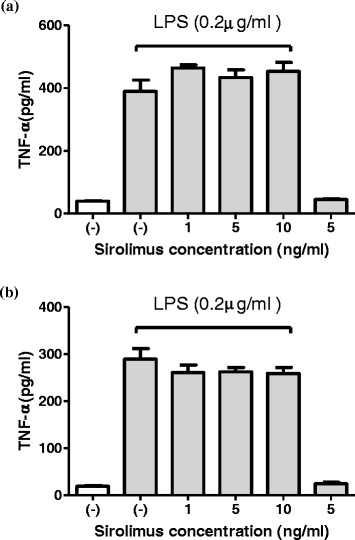
**Sirolimus did not suppress LPS-induced TNF-α production in THP-1 cells (a) and primary human monocytes (b).**

Sirolimus (1–10 ng/mL) significantly reduced the LPS-induced expression of MCP-1, RANTES, and IL-8 in the THP-1 cells (Figure 2a,c and e) and human primary monocytes (Figure 2b,d and f). In addition, Sirolimus (5 ng/mL) significantly reduced the LPS-induced expression of MIP-1α in the THP-1 cells (Figure 3a and c), whereas the expression of both MIP-1α and MIP-1β was reduced in LPS-treated human primary monocytes (Figure 3b and d). The data suggested that mTOR inhibition suppressed the expression of nephrotic-syndrome-related chemokines in the THP-1 cells and human primary monocytes. Sirolimus (1–10 ng/mL) did not significantly reduce the LPS-induced expression of TNF-i in THP-1 cells and human primary monocytes (Figure 4a and b).

### Sirolimus suppressed lipopolysaccharide-induced monocyte chemoattractant protein-1 expression through mitogen-activated protein kinase and nuclear factor-κB pathways in THP-1 cells

**Figure 5:**
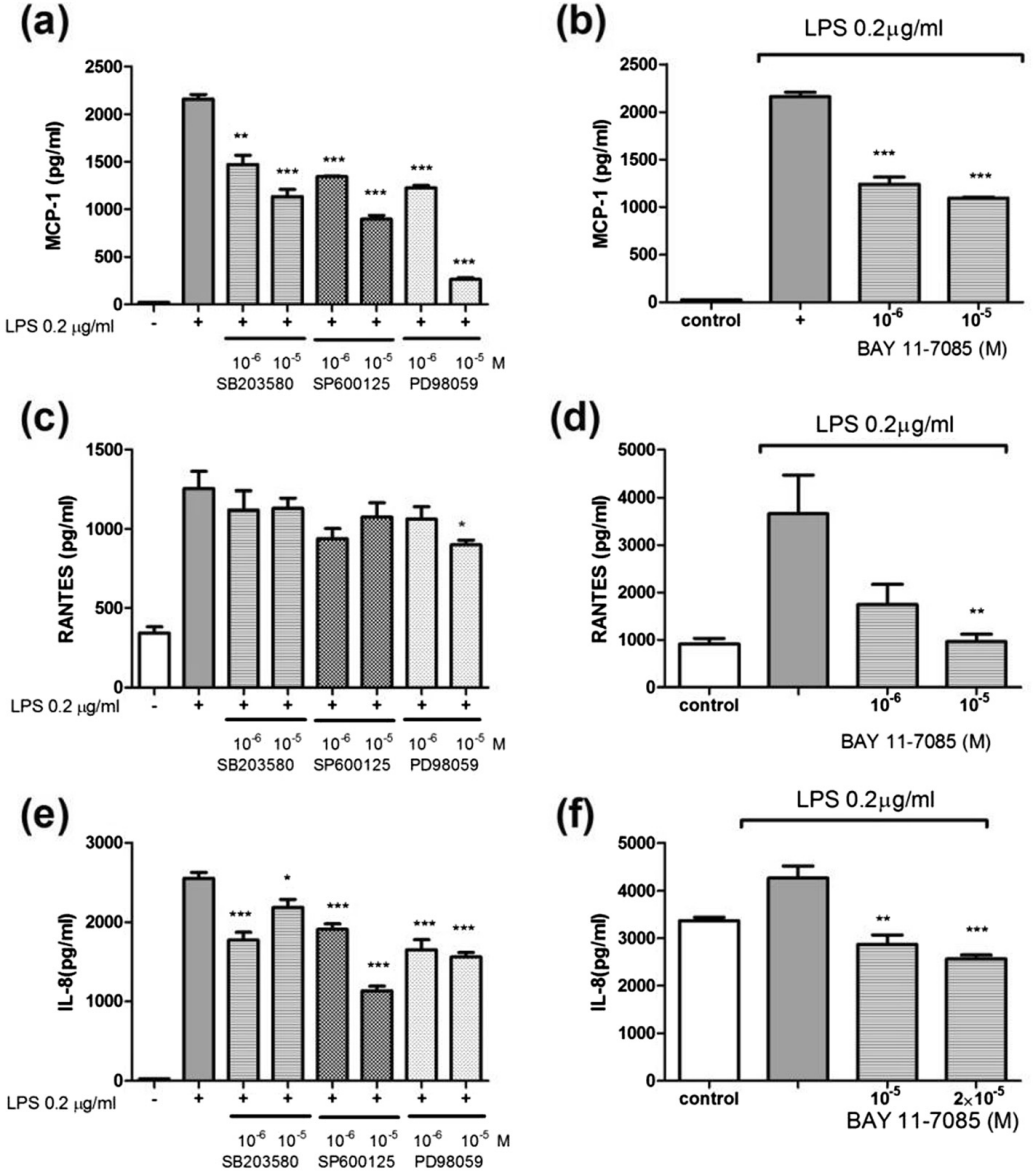
**PD98059 suppressed LPS-induced MCP-1 (a), RANTES (c), and IL-8 (e) expression.** SB203580 and SP600125 could only suppress LPS-induced MCP-1 and IL-8 expression. BAY 11–7085 suppressed LPS-induced MCP-1 **(b)**, RANTES **(d)**, and IL-8 **(f)** expression.

Figure 5a and e indicate that SB203580 (p38-MAPK inhibitor), SP600125 (JNK-MAPK inhibitor), and PD98059 (ERK-MAPK inhibitor) suppressed the LPS-induced expression of MCP-1 and IL-8, suggesting that MAPK signalling is involved in the LPS-induced expression of MCP-1 and IL-8 in THP-1 cells. Figure 5b, d, and f show that the NF-κB inhibitor, BAY 11–7085, significantly reduced the LPS-induced expression of MCP-1, RANTES, and IL-8 in THP-1 cells, signifying that NF-κB inhibitor signalling is involved in the LPS-induced expression of MCP-1, RANTES, and IL-8 in THP-1 cells.

**Figure 6:**
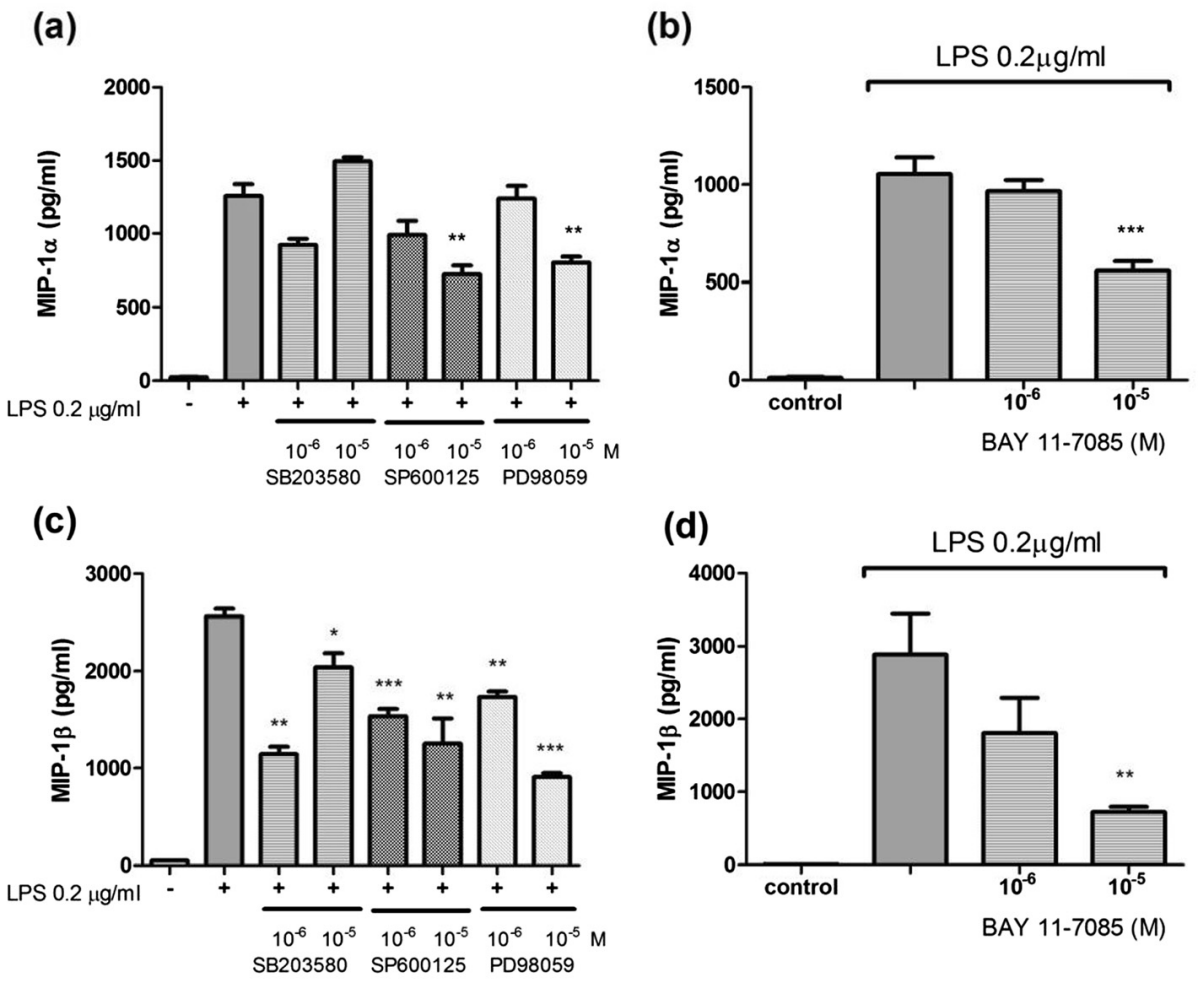
**SP600125 and PD98059 suppressed LPS-induced MIP-1α (a) and MIP-1β (c) expression.** BAY 11–7085 suppressed both LPS-induced MCP-1α and MCP-1β expression **(b, d)**. These results suggested that the p38, JNK-MAPKs, and NF-кB pathways were critical to LPS-induced MDC expression by monocytes.

As shown in Figure 6a and c, SP600125 and PD98059 reduced the LPS-induced expression of MIP-1α and MIP-1β in THP-1 cells; SB203580 suppressed the LPS-induced expression of MIP-1β, but did not reduce the expression of MIP-1α in THP-1 cells. Figure 6b and d show that BAY 11–7085 reduced the LPS-induced expression of MIP-1α and MIP-1β in THP-1 cells. Thus, both MAPK and NF-кB signalling are critical factors affecting the LPS-induced expression of MIP-1α and MIP-1β in THP-1 cells.

**Figure 7:**
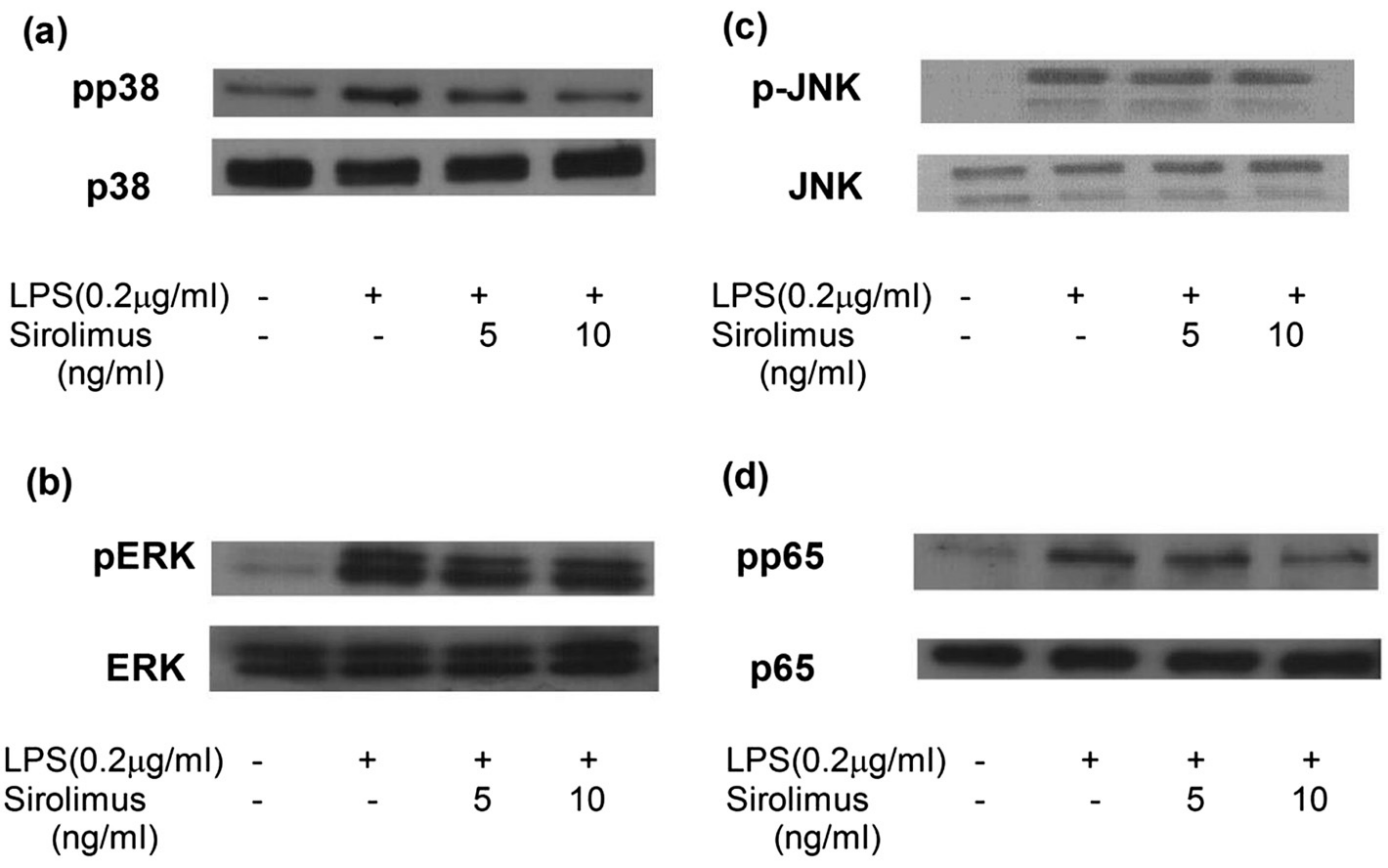
**Sirolimus suppressed LPS-induced pp65 and pp38 expression in THP-1 cells (a, b, c, d).**

In addition, sirolimus reduced the LPS-induced phosphorylation of p38 and p65 in human primary monocytes, but did not significantly affect the phosphorylation of JNK or ERK. This phenomenon indicates that sirolimus suppresses the expression of nephrotic-syndrome-related chemokines by modulating p38- and p65-mediated signalling pathways (Figure 7).

## Discussion

In this study, we demonstrated that the mTOR inhibitor suppressed chemokines, including MCP-1, RANTES, IL-8, and MIP- 1β in THP-1 cells, and MCP-1, RANTES, IL-8, MIP-1α, and MIP-1β in human primary monocytes. In addition, we determined that the suppressive effects of sirolimus in monocytes were mediated by the MAPK-p38 and NF-κB-p65 signalling pathways.

The immune system plays a crucial role in disease pathogenesis, evaluation, and treatment. With the signalling of chemokines and their corresponding receptors, monocytes gather in the target organ following injury and differentiate into macrophages and dendritic cells [12]. The inflammatory chemokine MCP-1 is a member of the cysteine-cysteine (CC) chemokine family [17]. In CCL2 (−/−) mice, neoplasms that grew failed to accumulate dendritic cell-like APCs in response to chemotherapy [18]. MCP-1 is also critical to the pathogenesis of atherosclerosis; considerable evidence has verified that the monocyte-containing MCPs and macrophage influence the growth of other cell types within the atherosclerotic lesion [19]. An increased level of MCP-1 expression in renal tissues is essential to monocyte/macrophage infiltration during the pathogenesis of renal injury [20]. In clinical applications, serum or urinary levels of MCP-1 could be markers of disease progression and treatment response [21,22]. The RANTES protein is also a member of the CC chemokine family. Previous studies have shown that increased expression of the RANTES protein 3 to 5 d after the activation of T cells facilitated leukocyte infiltration and increased the duration of the inflammatory response [23]. The RANTES and its receptor have been detected in various hematological malignancies and lymphomas and in many solid tumors. Inhibiting the binding of RANTES to its receptor or the secretion of RANTES is a new chemotherapy strategy [24]. A previous study suggested that the expression of RANTES in the cerebral microcirculation of patients with Alzheimer’s disease is elevated, and that oxidative stress upregulated RANTES expression in rat brain endothelial cells [25]. Another study determined that the expression of the MCP-1 and RANTES proteins by tubular epithelial cells correlated with proteinuria and was associated with renal interstitial cell infiltration and fibrosis [26]. Manipulating the expression of RANTES might facilitate a beneficial treatment strategy for various diseases, including cancer, dementia, and renal diseases [27]. The plasma level of IL-8 was significantly higher during nephrotic-syndrome relapse than during remission [28]. IL-8 and IL-17 enhance the activity of matrix metalloproteinase-2 and −9 (MMP-2,-9) which in turn increase the metastatic activity of the underlying malignancy [29]. IL-8 and other chemokines have been considered to play a role in developing peripheral artery disease [30]. Macrophage inflammatory markers (MIP-1α, β) have been determined to be critical factors affecting atherosclerosis [31,32]. A previous study suggested that MIP-1α and β were expressed by infiltrating leukocytes, the renal tubular cells, and peritubular capillaries in patients with kidney diseases [33].

mTOR is a component of two major intracellular signalling complexes (mTORC1 and mTORC2) that play dissimilar roles downstream. mTORC1 is activated by growth factors and amino acids and controls cellular proliferation, promoting processes such as DNA translation, RNA transcription, ribosomal biogenesis, and cell cycle progression [34]. Rapamycin is an alternative immunosuppressive treatment choice of calcineurin inhibitors used to treat chronic allograft damage [35]. Currently, mTOR inhibitors have been applied to treat several types of illnesses, including cancer, arteriosclerosis, and autoimmune diseases; however, numerous proinflammatory side effects have been observed, including interstitial pneumonitis, glomerulonephritis with proteinuria, lymphocytic alveolitis, and anemia [36-39]. Weichhart et al. determined that the mTOR inhibitor upregulated IL-12 production in innate immune cells, such as monocyte/macrophages, through the transcription factor NF-kB, but blocked the release of interleukin-10 through the transcription factor STAT3 [40]. mTOR inhibitors could also induce macrophage apoptosis in M2 phase rather than in M1 phase [41]. These results were contributed to understanding inflammatory conditions of mTOR inhibitors, and facilitated new therapeutic options. The role of mTOR inhibitors in the secretion of chemokines by mononuclear cells requires further evaluation.

In this study, we determined the suppressive effect mTOR inhibitors exert on chemokines secreted in cell models and human primary monocytes. The results indicated that mTOR inhibitors may facilitate therapeutic clinical treatments. In addition, we investigated the intracellular signal pathway to explore the detailed mechanism by which suppression occurred. The NF-κB-, ERK-, and p38-mediated activation of MAPK signal transduction pathways is critical to the inflammatory response [42,43]. The suppressive effect sirolimus exerts on the expression of LPS-induced phosphorylation of p38 and p65, but not of JNK or ERK, suggested that the mTOR inhibitor suppressed the expression of chemokines by modulating the p38- and p65-mediated signalling pathways. The immunosuppressive effect of glucocorticoids occurred because of the MAPKs [44]. The calcineurin inhibitors cyclosporine and tacrolimus reduce the responses of NF-κB activation and therapeutically regulate the expression of MAPKs [45], and mycophenolate mofetil inhibits the phosphorylation of NF-κB and JNK, and is a possible alternative treatment [46]. Our results suggested that mTOR inhibitors suppress the expression of chemokines by inhibiting the NF-κB-p65 and MAPK-p38 signalling pathways in monocytes. Further pathway investigation may be necessary.

### Limitations

Certain limitations to our findings must be considered. We evaluated the suppressive effects sirolimus exerted on the expression of monocyte-secreted chemokines in cell models. In future studies, primary monocytes can be collected from patients with diseases to investigate the effect of mTOR inhibitors and verify our findings.

## Conclusions

An mTOR inhibitor, sirolimus, downregulated the expression of chemokines, including MCP-1, IL-8, RANTES, MIP-1α, and MIP-1β, by inhibiting the NF-κB-p65 and MAPK-p38 signalling pathways in monocytes. These results indicated that mTOR inhibitors can be used in treatments for inflammatory diseases. Future studies including larger patient numbers are necessary.
